# The Tertiary Origin of the Allosteric Activation of *E. coli* Glucosamine-6-Phosphate Deaminase Studied by Sol-Gel Nanoencapsulation of Its T Conformer

**DOI:** 10.1371/journal.pone.0096536

**Published:** 2014-05-02

**Authors:** Sergio Zonszein, Laura I. Álvarez-Añorve, Roberto J. Vázquez-Núñez, Mario L. Calcagno

**Affiliations:** Departamento de Bioquímica, Facultad de Medicina, Universidad Nacional Autónoma de México, Ciudad Universitaria, Mexico City, Mexico; Tulane University, United States of America

## Abstract

The role of tertiary conformational changes associated to ligand binding was explored using the allosteric enzyme glucosamine-6-phosphate (GlcN6P) deaminase from *Escherichia coli* (EcGNPDA) as an experimental model. This is an enzyme of amino sugar catabolism that deaminates GlcN6P, giving fructose 6-phosphate and ammonia, and is allosterically activated by *N*-acetylglucosamine 6-phosphate (GlcNAc6P). We resorted to the nanoencapsulation of this enzyme in wet silica sol-gels for studying the role of intrasubunit local mobility in its allosteric activation under the suppression of quaternary transition. The gel-trapped enzyme lost its characteristic homotropic cooperativity while keeping its catalytic properties and the allosteric activation by GlcNAc6P. The nanoencapsulation keeps the enzyme in the T quaternary conformation, making possible the study of its allosteric activation under a condition that is not possible to attain in a soluble phase. The involved local transition was slowed down by nanoencapsulation, thus easing the fluorometric analysis of its relaxation kinetics, which revealed an induced-fit mechanism. The absence of cooperativity produced allosterically activated transitory states displaying velocity against substrate concentration curves with apparent negative cooperativity, due to the simultaneous presence of subunits with different substrate affinities. Reaction kinetics experiments performed at different tertiary conformational relaxation times also reveal the sequential nature of the allosteric activation. We assumed as a minimal model the existence of two tertiary states, *t* and *r*, of low and high affinity, respectively, for the substrate and the activator. By fitting the velocity-substrate curves as a linear combination of two hyperbolic functions with *K*_t_ and *K*_r_ as K_M_ values, we obtained comparable values to those reported for the quaternary conformers in solution fitted to MWC model. These results are discussed in the background of the known crystallographic structures of T and R EcGNPDA conformers. These results are consistent with the postulates of the Tertiary Two-States (TTS) model.

## Introduction

The concept of allostery, introduced by Monod et al. in 1963, embodies one of the fundamental principles of life [1]. Allosteric effects are a consequence of the thermodynamic coupling of a pair of binding sites in a protein molecule that are distant and have different functional properties. The functional coupling between binding sites is mediated by specific conformational changes in the protein molecule and were the object of a classic thermodynamic analysis by Wyman in 1948 with the name of linked functions [2]. Oligomeric proteins with identical thermodynamically-linked sites located in different subunits give rise to homotropic cooperativity, which frequently is incorporated into the concept of allostery. Indeed, both properties appear frequently associated and are considered as two aspects of the same phenomenon. Monod et al. in their landmark model presented a unified theory of cooperativity and heterotropic allosteric effects [3]. Their model conceived allosteric transitions as symmetric quaternary rearrangements of subunits between two extreme conformational states T and R, with different binding affinities (MWC model [3]). The postulates of MWC model were supported along the subsequent years by many structural and physicochemical evidences, and several allosteric proteins were crystallized in either quaternary conformational state, foreseen by the model. In most cases, the comparison of the crystallographic structure of the extreme allosteric conformers, in spite of their limitations, afforded the first descriptions of the mechanism of allosteric transitions [4]. Later studies on hemoglobin and other well-known allosteric proteins demonstrated that the MWC model alone was not sufficient to explain the tertiary-quaternary coupling of human hemoglobin. The evidence that the tertiary conformational changes alone can produce heterotropic, that is, allosteric effects in hemoglobin contradict the original postulates of the MWC model [5]. From these and other experimental evidences, Henry et. al. proposed a MWC-inspired theory to account for the role of tertiary allosteric transitions and its coupling to the quaternary transition in hemoglobin [6]. Their model, designated TTS for tertiary two states, considers that the oxygen affinity of each hemoglobin subunit is determined only by its tertiary conformational state, while cooperativity is of quaternary origin. The tertiary-quaternary coupling is explained by the mutual biasing between the tertiary and quaternary conformational states, which undergo sequential and concerted transitions respectively.

The dissociation of heterotropic allostery and cooperativity has been observed under diverse experimental conditions, in other allosteric proteins as fructose 1,6-bisphosphatase by chemical modification [7] or aspartate transcarbamoylase, by site-directed mutagenesis [8]. Natural biodiversity offers a rich variety of allosteric mechanisms, thus stressing the importance of their study in different experimental models. The present research takes as a model the enzyme glucosamine-6-phosphate deaminase from *Escherichia coli* (EcGNPDA, E.C. 3.5.99.6) [9]–[11], with the scope of characterizing the linkage between its subunit local mobility and its allosteric kinetics. This enzyme has been subject of many structural and physicochemical studies with emphasis in its allosteric properties. EcGNPDA isomerizes and deaminates glucosamine 6-phosphate (GlcN6P), forming fructose 6-phosphate and ammonia and it is allosterically activated by *N*-acetylglucosamine 6-phosphate (GlcNAc6P). It is a homohexameric enzyme, displaying homotropic cooperativity with respect to GlcN6P and *K*-type allosteric activation by GlcNAc6P [11]. Our knowledge of EcGNPDA allosteric properties derives from the analysis of the crystallographic structures of its extreme conformational states, T (PDB 1FSF) and R (PDB1FS5) [12]-[14] and from physicochemical studies of the wild-type enzyme and some chemically or genetically modified forms [15]–[17]. In previous research on EcGNPDA we succeeded to suppress cooperativity while keeping allosteric activation by interfering with intersubunit contacts by chemical modification or mutagenesis [18], [19]. Crystallographic studies by Rudiño *et al*. provided experimental support for the existence of tertiary structural sub-states of EcGNPDA R conformer. Some other studies revealed the existence of local intrasubunit changes accompanying the quaternary transition of EcGNPDA and the apparent uncoupling of allostery and cooperativity [20], [21]. The lack of information about the time-course of these structural changes makes difficult the discussion of their role in EcGNPDA allosteric mechanism.

Nanoencapsulation of EcGNPDA in silica sol-gels improved the understanding on its allosteric mechanism, allowing us to characterize its transitional short-lived intermediates. Silica polymers formed surrounding the protein molecules, generate a wet nanostructured environment that restricts the protein conformational mobility. This approach has proven to be a valuable tool for the study of allosteric proteins, particularly human hemoglobin [22]–[24] and allosteric enzymes [25], [26]. Under our experimental conditions, we succeeded to block the quaternary transition of gel-trapped EcGNPDA, its catalytic and allosteric functions were conserved and its intrasubunit mobility was considerably slowed down. This approach gave us experimental access to the otherwise undetectable conformational sub-states of the allosteric activation process by GlcNAc6P. We centered our study on the activation of the T state, which in the case of the soluble enzyme is the most populated conformation in absence of ligands. This experimental approach gave us access to the otherwise undetectable conformational sub-states of the allosteric activation process of this enzyme.

## Materials and Methods

### Biochemicals

Most biochemicals were purchased from Sigma-Aldrich S.A. de C.V. (Mexico) or were reagent-grade products. GlcNAc6P was synthesized according to the procedure by Leloir and Cardini [27]. All buffer solutions used had their pH adjusted at 20°C.

### Wild-type enzyme and site-directed mutants

EcGNPDA and its mutant forms were obtained by a single step purification procedure using allosteric-site affinity chromatography on a matrix of *N*-6-aminohexanoyl-glucosamine 6-phosphate agarose [19]. Specific elution was performed with 0.01 M sodium GlcNAc6P in 0.1 M Tris-HCl buffer pH 7.8.

### Construction of a fluorescent allosteric reporter

A mutant form of EcGNPDA was designed to introduce a fluorescent reporter signal for monitoring the molecular conformational changes. On a previously constructed triple mutant form of EcGNPDA in which all the surface-exposed cysteine residues were replaced by serine (Cys118-Ser:Cys228-Ser:Cys239-Ser) [28], we introduced the additional replacements Asp165-Cys and Ser206-Trp. The site-directed mutation was carried out by oligonucleotide-directed mutagenesis using the QuickChange-II kit (Agilent Technologies, U.S.A.) following the instructions of the manufacturer. A pTZ18R*nagB* plasmid containing the mutant form of *nagB* gene [29] was used to transform the *E. coli* strain IBPC590 Δ*nag*, previous verification of the plasmid insert by DNA sequencing. Along all the purification procedure and the experimental work with this mutant, the thiol group of the introduced Cys165 was maintained in its reduced state by working in the presence of 10 µM Tris(2-carboxyethyl)phosphine.

The kinetic and allosteric properties of this mutant (Mutant F) are not significantly different to those of the wild-type enzyme (Table 1). The rationale for the selection of these positions comes from the comparison of the crystallographic structures of EcGNPDA using computational energy minimizations and short molecular dynamic simulations of both quaternary conformers. This comparison shows a substantial shortening of the distance between the residues in positions 165 and 206 during the transition from T to R state [21]. The involved residues are located in two subunits without permanent interfacial contacts. On the basis of this observation, we replaced for a cysteine and a tryptophan the residues at the selected positions to form a fluorescence quenching pair. We observed the quenching of tryptophan emission at 334 nm in response to GlcNAc6P binding to the soluble enzyme form. The binding curve of the soluble mutant F resulting from fluorescence measurements, display homotropic cooperativity (not shown). Contrastingly, the fixed enzyme in the T quaternary conformation shows quenching of tryptophan fluorescence emission at 320 nm upon activator binding, producing hyperbolic saturation curves.

**Table 1:** Reference values for the wild-type enzyme [20] and for the mutant F in solution.

Enzyme	*K*_T_^a^ (mM)	*K*_R_^a^ (mM)	*k*_cat_^a^ (s^−1^)	*c* ^a^	*L* ^a^	*h* ^b^
Wild-Type	22.0±2.0	0.55±0.05	158±8	0.025±0.002	10^6^±1.9×10^4^	2.9±0.1
Mutant F	7.86±0.59	0.44±0.01	107±1	0.056±0.004	10^5^±10^3^	2.0±0.1

a Parameters obtained by fitting velocity against substrate concentration data to the MWC general equation [6].

b Obtained by fitting velocity data to Hill equation.

### Enzyme-doped gels

We encapsulated EcGNPDA by alkaline polymerization of tetramethyl-*o*-silicate in the presence of the enzyme, according to the procedure described by Bettati *et al.* [30], except that the protein dialysis buffer used was 0.1 M Tris-HCl at pH 7.5. The final enzyme concentration in the sol-gels was between 5 and 20 µM. We produced thin gel monoliths of nearly 100 µm-thick and with a total volume of 100 µL, layered on quartz windows of 38×9.15×1 mm. The monoliths have good optical quality and adequate thickness and porosity for the rapid diffusion of ligands [30]. The absence of light scattering produces gels of crystal clear aspect, suggesting that their pores are smaller than 100 Å [31]. Thin gel monoliths were kept in 10 mM Tris-HCl buffer pH 7.0 at 4°C (storage buffer) before use. The gels were ready for use 48 h after their preparation. Ligand-free EcGNPDA was used to produce T-gels; this enzyme is expected to be in an all-T state according to its known allosteric properties [16]. Gels containing the deaminase in the R state were initially prepared from gelation mixtures containing 2 mM GlcNAc6P and conserved in the storage buffer added with the same concentration of allosteric activator. This concentration is 70 times the *K*_dis_ value for the ligand [16]. Ligand-free R gels (R_0_) were prepared just before use, by overnight dialysis against 10^4^ volumes of storage buffer to get rid of the activator. We used R_0_ gels in this research only for reference experiments. We used the following notation to represent the different conformational and binding states considered in this research. The quaternary fixed conformations T or R followed by a zero subscript represents the ligand-free enzyme, and the A subscript indicates saturation with GlcNAc6P (*i.e.* T_0_, T_A_, R_0_ and R_A_). The low and high-affinity tertiary states are indicated as *t* and *r*, respectively (as in T_0_ *t*, R_A_ *r*, etc.).

### Circular dichroism spectra

CD spectra in the aromatic absorption range were recorded with a Jasco J-715 spectropolarimeter using 5 mm light-path quartz cuvettes filled with 100 mM Tris-HCl buffer pH 7.7 (30°C). The enzyme-doped gel monoliths were immersed with the quartz support adhered to photomultiplier wall of the cuvette. The temperature of the sample compartment was set at 30°C.

### Kinetics of the catalyzed reaction

The enzyme used in the kinetic experiments was prepared by disintegration of the enzyme-doped gels at low-power sonication, as described by Pioselli *et al*. [32]. The particle suspension was washed several times with storage buffer and decanted by gravity to discard the enzyme leaked from the gel during sonication. The protein concentration in the particulate suspension was measured after depolymerization of the gel in 1 M NaOH at room temperature followed by intense vortexing until getting a clear solution. The concentration of EcGNPDA in the initial suspension was obtained from its absorbance at 290 nm and a molar absorptivity of 6.3×10^5^ M^−1^ cm^−1^. This value was experimentally determined from an EcGNPDA solution of known concentration dissolved in 1 M NaOH. GlcN6P deamination rate was measured at 30°C in 100 mM Tricine buffer, pH 7.8 (assay buffer). The amount of fructose 6-phosphate formed at a fixed time was measured by using a previously described procedure [11], always keeping the reaction progress below five percent of substrate conversion. The reaction was started by the addition of a vortexed suspension of micro-disaggregated gel to get a final enzyme concentration of 2 nM in the microparticulate suspension and added to the assay mixture in a shaking bath.

### Data analysis

Data fitting and graph plotting were processed with Prism 5.0 for MacOS X (GraphPad Software Inc., U.S.A.). Non-linear regression analyses were always checked by inspection of their residual plots. SD values given in tables and text are the standard deviation of the regressions.

### Fluorometric monitoring of conformational changes

We recorded the fluorescence emission spectra on a PC-1 spectrofluorometer, ISS (Champaign, IL, USA). The cuvettes were filled with the assay buffer and kept at 30±0.1°C. Mutant F doped monoliths were placed in a triangular prismatic quartz cuvette (Starna 4-SB-Q-10 cell), the monolith on the gel-supporting window was placed on the oblique wall of the cuvette, such as both excitation and emission beams pass through the solvent. The rate of tertiary transitions was determined by the time-course of tryptophan fluorescence quenching, measured by exciting at 295 nm and recording the emission at 320 nm. The relaxation process was started by the addition of variable concentrations of allosteric activator.

## Results and Discussion

### Kinetics of the reaction catalyzed by EcGNPDA nanoencapsulated in the T conformation

**Figure 1:**
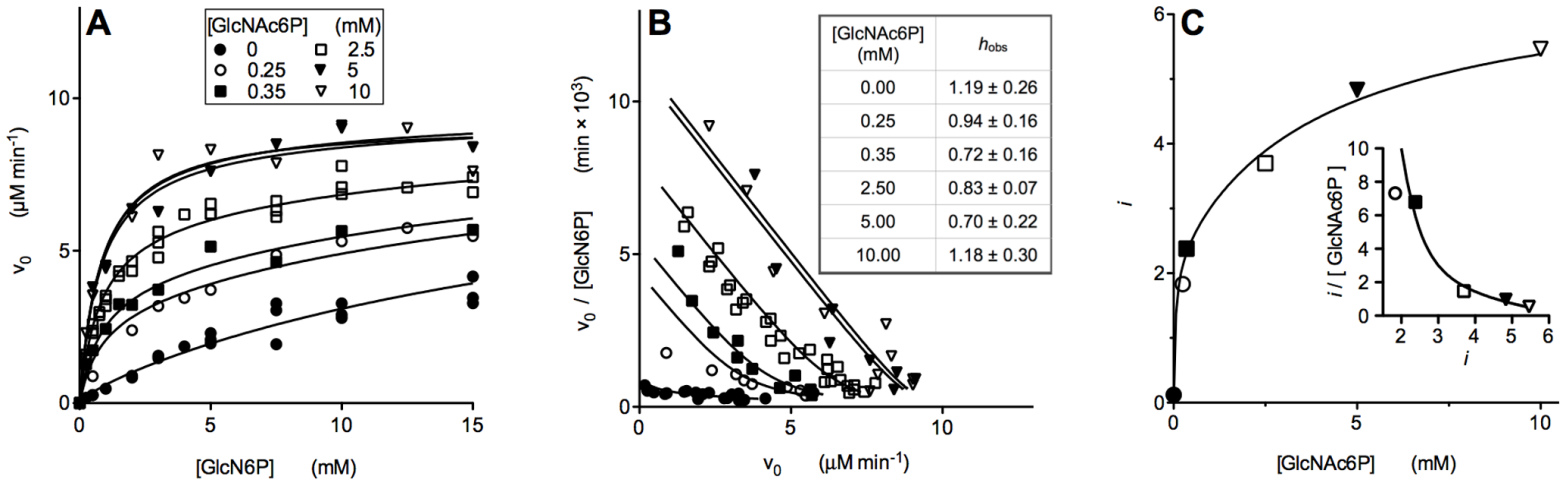
Kinetics of the reaction catalyzed by EcGNPDA nanoencapsulated in the T conformation at variable GlcNAc6P concentration. Curves of velocity against substrate concentration, obtained before the tertiary transition reached the conformational equilibrium. The presence of transient intermediates containing subunits with low or high affinity for the GlcN6P is apparent. Substrate concentration was varied over a wide concentration range to detect binding by both tertiary conformers. The enzyme concentration was 2(A) Velocity *versu*s substrate plots taken at different allosteric activator concentrations. Data were fitted by global non-linear regression to equation 1. The symbols representing each activator concentration are equal in the three panels. (B) Scatchard transform of data in panel A. The plotted lines correspond to the simulated function using the parameters obtained from the global regression; negative cooperativity is apparent at intermediate GlcNAc6P concentrations. The Hill equation was used as a diagnostic tool for negative cooperativity and this panel shows the corresponding Hill coefficients. (C) Parameter *i* obtained from fitting data in panel A to equation 1, plotted against GlcNAc6P concentration. This parameter value describes the change in subunit tertiary composition with increasing activator concentration; it represents the number of subunits per molecule in the *r* state. Inset: Scatchard plot, showing the apparent negative cooperativity of GlcNAc6P binding. The existence of at least two different affinity states for the allosteric activator can be appreciated.

**Figure 2:**
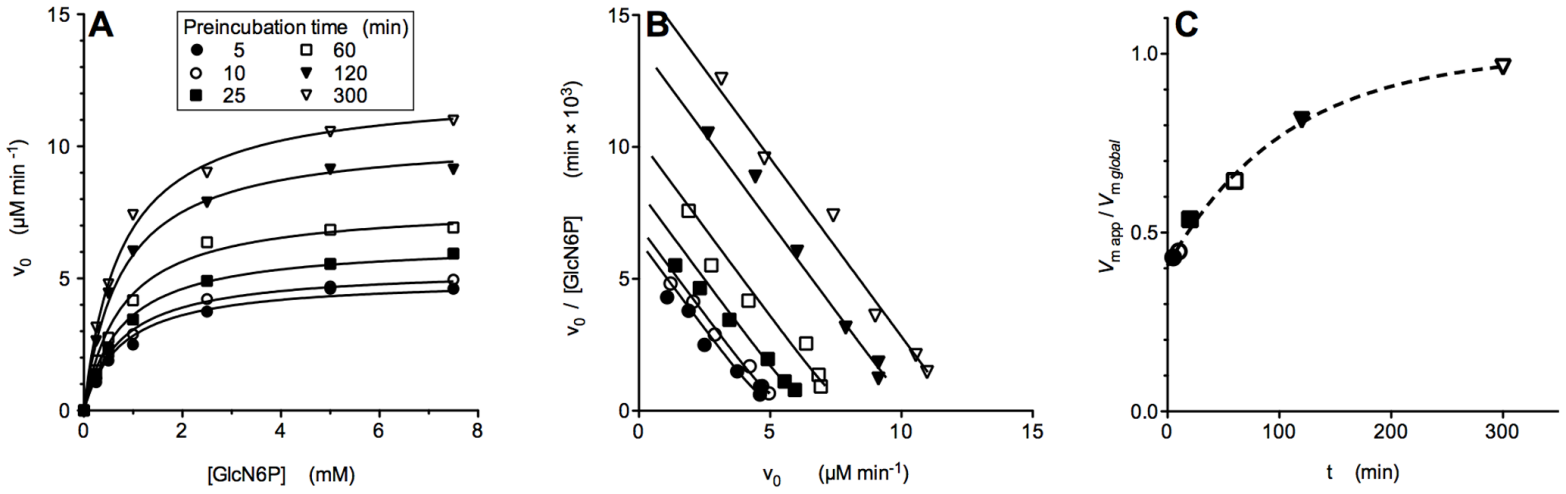
Time-course of the tertiary conformational relaxation of the nanoencapsulated T-conformer, analyzed through the catalytic activity of EcGNPDA. (A) Plot of velocities against substrate concentrations at a fixed (0.3 mM) activator concentration. Substrate concentration was varied in an interval far below the *K*_t_ value for the enzyme in the T state (Table 2), to mainly reveal the activity of the high affinity subunits. Data were fitted to hyperbola. The enzyme concentration was 2 nM. The symbols representing each incubation time are equal in the three panels. (B) Scatchard plot of the same data. Note the absence of homotropic cooperativity and the time-dependent recruitment of subunits in a high affinity state. The plotted lines were obtained by simulating the corresponding fitted transform. (C) Time-course of the allosteric activation of the T conformer, appreciated as an exponential increase of the apparent *V*_max_. Data were fitted to the first order equation yielding a *k*_obs_ at 0.3 mM GlcNAc6P of 1.6×10^−4^±0.2×10^−4^ s^−1^. Note that along the assay, the enzyme was already in contact with GlcNAc6P; for this reason the allosteric activation is in progress before starting the enzyme assay. Due to this experimental constraint, the observed span of fractional *V*_max_ values is lesser than one (Figure 2C), even when referred to the extrapolated value at zero time.

A general finding of all the kinetic experiments with EcGNPDA nanoencapsulated in T quaternary conformation is the absence of positive cooperativity for the substrate. The plot of initial velocities against substrate concentration for the encapsulated EcGNPDA in T state shows hyperbolic kinetics at zero (T_0_) and at saturating GlcNAc6P concentrations (T_A_) (Figure 1A). Apparent negative cooperativity for the substrate was found at intermediate concentrations of activator, evidenced by Hill coefficients lesser than one. This effect can be explained by the simultaneous presence of subunits of low and high substrate-affinity (Figure 1B). The observed *t-r* ratio depends on GlcNAc6P concentration and is not modified by the substrate. The latter effect is a consequence of the suppression of the homotropic cooperativity. The progress of the *t-r* transition depends on the time of contact of the enzyme with the allosteric activator, which was kept constant at 10 min, corresponding to the duration of our standard activity assay. This tertiary transition is slowed down by nanoencapsulation, as it will be shown in the following section (Figure 2). The velocity *versus* substrate curves shown in Figure 1 represent “kinetic snapshots” of the progress of the *t-r* transition as a function of the allosteric activator concentration, which is appreciated by the change in the tertiary heterogeneity of the enzyme. We analyzed this set of curves (Figure 1) by global non-linear regression with the following semi-empirical expression:
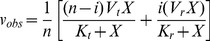
(1)


This equation gives the sum of the reaction rates contributed by each tertiary conformer, *v*_obs_, as a function of X, the ligand concentration. It derives from a minimal model assuming the existence of two tertiary states with different substrate affinities; the expression is the linear combination of two hyperbolic binding steps. The *K*_M_ values for the subunits in their low and high-affinity are, respectively, *K*_t_ and *K*_r_, *n* is the number of subunits, the parameter *i* is the number of sites in the high-affinity tertiary state per oligomer and *n-i* is the corresponding number of subunits in the low-affinity state. Sidewise, the fraction of subunits in the high affinity state, *i/n*, is a tertiary equivalent to the function of state defined by Monod *et al*. for the quaternary states [3]. From the global non-linear regression of this experiment to equation 1, we obtained the values for the *K*_t_ and *K*_r_. The fitted values for these constants are in good agreement with the MWC substrate dissociation constants *K*_R_ and *K*_T_, reported for the enzyme in solution (Tables 1 and 2). This observation indicates that nanoencapsulation does not significantly perturb the binding properties of EcGNPDA. The *k*_cat_ values were determined by using the *V*_t_ and *V*_r_ parameters. The *k*_cat-t_ value is similar to the *k*_cat_ of the soluble enzyme (Tables 1 and 2). This is the expected result for an enzyme that behaves as a perfect *K*-system in solution. However, the nanoencapsulated enzyme shows a *k*_cat-r_ greater than *k*_cat-t_, thus revealing an implicit *V*-effect. The *r* subunits must be functionally different according to the quaternary state of the molecule. Accordingly, the gel-trapped T_A_r state results catalytically more efficient than the activator saturated enzyme in solution (R_A_r). This is the first demonstration of the properties of this transient species, T_A_r, which was stabilized by nanoencapsulation.

**Table 2:** Kinetic constants for the deamination reaction of the nanoencapsulated T conformer of EcGNPDA.

*K*_t_ (mM)^a^	*K*_r_ (mM)^a^	*K*_r_ (mM)^b^	*k*_cat-t _(s^−1^)^a^	*k*_cat-r _(s^−1^)^a^
23.1±3.6	0.98±0.06	0.76±0.02	185±16 s^−1^	279±27 s^−1^

a From experiments shown in Figure 1; parameters were obtained from global data fitting to equation 1.

b From experiment in Figure 2 that detects the catalytic activity of the high-affinity tertiary state. The *K*_r_ value is obtained from fitting to hyperbola.

The *i* parameter is a useful analytical tool to estimate the tertiary conformational landscape, giving a picture of the enrichment of the enzyme population in *r* state subunits (Figure 1C). The inset of this figure shows a Scatchard-like replot that demonstrates the apparent negative cooperativity for the allosteric effector. This finding suggests the existence of two tertiary conformers with different binding affinities for GlcNAc6P.

### The T_0_t →T_A_r transition of EcGNPDA analyzed through its catalytic activity. Targeting the activity of r subunits

It is known that nanoencapsulation slows down tertiary relaxations in hemoglobin [24]. The time course of the tertiary conformational relaxation of EcGNPDA in T_0_t conformer upon GlcNAc6P binding was directly explored by the kinetics of its catalyzed reaction. This experiment consists of a series of reaction rate measurements performed on samples previously incubated with a fixed concentration of GlcNAc6P for different times. The activator concentration was 0.3 mM, chosen to saturate the *r* state. Each curve obtained corresponds predominantly to the activity of the *r* subunits. We performed the reaction rate measurements by varying the substrate concentration ranging from 0 to 7.5 mM. The set of curves of velocity against substrate concentration in Figure 2A, illustrates the time dependence of the tertiary *t-r* transition involved in the allosteric activation of EcGNPDA. The observed kinetics is largely hyperbolic, with a globally fitted *K*_M_ value of 0.76±0.02 mM and increasing apparent *V*_max_ values. This *V*-effect evidences the sequential recruitment of *t* subunits towards the *r* state. The experiment starts with a molecular population rich in the ligand-free, low-affinity state, T_0_ *t*. Its contribution to the measured reaction rates is minor and negligible in mostly all of the data set. Taking into consideration the obtained *K*_t_ and *K*_r_ values from the previous experiment (Table 2), we can exclude data points with the largest error due to the presence of *t* conformers, without a significant impact on the apparent *V*_max_ values fitted. The variation of these apparent values as a function of time, reports the tertiary conformational relaxation; velocity increases as the population gets enriched in *r* conformers, which are mostly the dominant species at the substrate concentration range explored. We can analyze this enrichment in *r* subunits, which follows apparent first-order kinetics with a *k*_obs_ of 1.6×10^−4^±0.2×10^−4^ s^−1^ (Figure 2C). A detailed kinetic analysis of this conformational relaxation by means of a direct fluorometric method is presented in the following section.

### Fluorometric study of the relaxation kinetics of the nanoencapsulated T conformer of EcGNPDA

**Figure 3:**
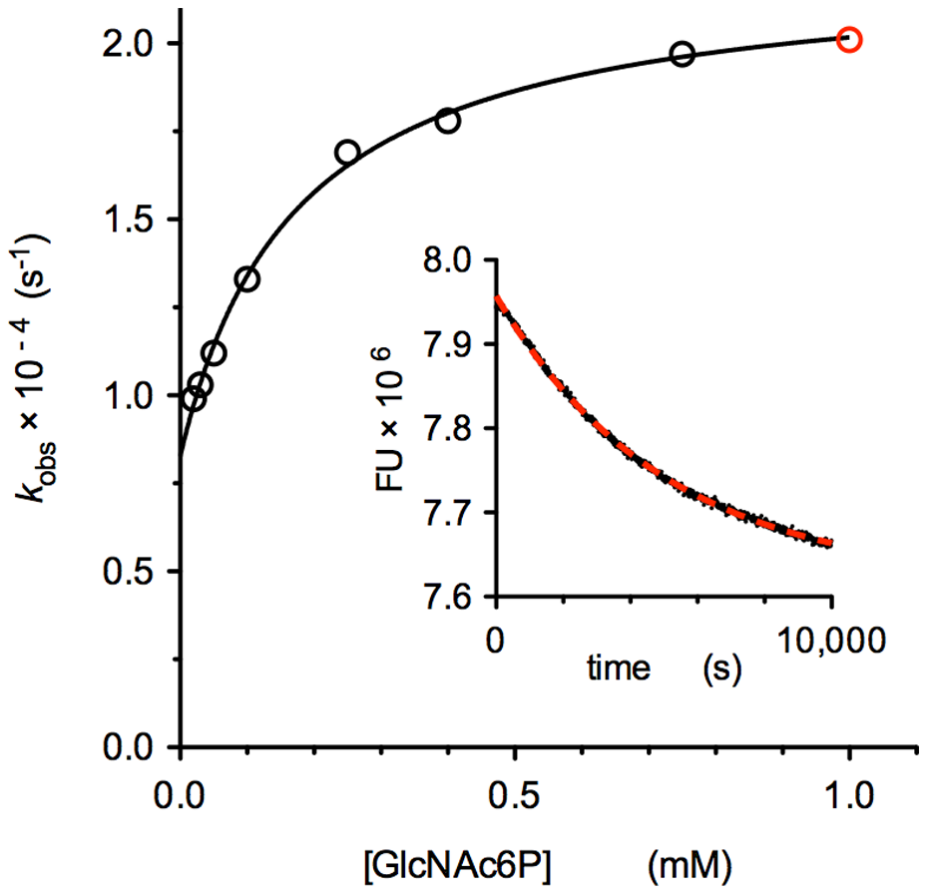
Relaxation kinetics of the nanoencapsulated F mutant in T conformation: effect of GlcNAc6P concentration. The florescence decay curves recorded at different activator concentrations were fitted to a single exponential function and the obtained *k*_obs_ values were replotted as a function of the allosteric activator concentration. Data were fitted to equation 2. The resulting rate constants are shown in Table 3. The inset shows an example of one of the recorded relaxation curves fitted to the first-order equation (dashed red line). This example corresponds to the highest GlcNAc6P concentration tested (1 mM) (red symbol).

**Figure 4:**
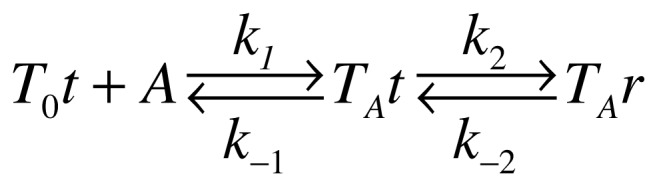
Proposed two-step mechanism. The scheme depicts the mechanism with a fast step of activator binding, followed by a slow tertiary transition.

Wild-type EcGNPDA lacks of an adequate endogenous spectral signal for the kinetic study of its conformational transitions. For this purpose, we designed a multiple mutant form of EcGNPDA described in the methodological section (mutant F), whose intrinsic fluorescence is quenched by GlcNAc6P binding. The relaxation kinetics of the ligand-free enzyme encapsulated in the T state was determined at different GlcNAc6P concentrations, and the resulting *k*_obs_ were plotted against the ligand concentration. The plot of the calculated *k*_obs_ as a function of GlcNAc6P concentration is hyperbolic with a positive ordinate at the origin (Figure 3). This result is consistent with the following two-step mechanism, with a first rapid binding step followed by a slower conformational transition (Figure 4). The bimolecular step is expected to be the faster one; the unimolecular step, the rate-limiting step, involves a slower conformational change, further slowed down by encapsulation. This step is certainly the origin of Trp206 fluorescence quenching, given the geometry of the involved residues in the hexamer. This analysis is consistent with an induced-fit mechanism triggered by GlcNAc6P binding. The corresponding function relating *k*_obs_ with activator concentration, derived from the mechanism in the scheme shown in Figure 4, is [33]:
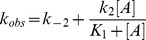
(2)


Where [A] is GlcNAc6P concentration, *K*_1_ is the dissociation constant for the ligand. The fitted parameters to this equation are shown Table 3. The overall apparent dissociation constant for this process, 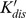, is given by the following expression [33]:
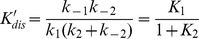
(3)


**Table 3:** Relaxation kinetics parameters of the nanoencapsulated ligand-free T conformer of mutant F, upon binding of GlcNAc6P at variable concentrations.

*k*_2_^a^	*k*_−2_^a^	*K*_1_^a^	*K′*_dis_^b^
8.6×10^−5^±0.4×10^−5^ s^−1^	1.30×10^−4^±0.05×10^−4^ s^−1^	19.3×10^−5^±7.0×10^−5^ M	7.7×10^−5^±2.8×10^−5^ M

a Parameters obtained by fitting data in figure 3 to equation 2.

b Calculated from equation 3.

Where *K*_2_ = *k*_−2_/*k*_2_

The binding properties of mutant F were verified in solution, showing a close resemblance to the activator binding curve of the wild type enzyme (not shown). Also, the apparent first-order rate constant (*k*_obs_) resulting from the present relaxation kinetics with mutant F (inset of Figure 3), is similar to the calculated *k*_obs_ from data in Figure 2C. These observations support the reliability of relaxation experiments using this mutant.

### The tertiary origin of allosteric activation and the abolition of quaternary transition demonstrated by CD spectrometry

Nanoencapsulated EcGNPDA has its quaternary transition abolished, as indicated by the loss of homotropic cooperativity. In additional support of this conclusion, we measured the CD spectral changes associated to GlcNAc6P binding, as a diagnostic tool for the quaternary state.

The T-R transition of soluble EcGNPDA produces a well-characterized ellipticity change at 274 nm, which has been recognized as a specific reporter signal for the allosteric transition [34], [35]. We took advantage of this property for monitoring the quaternary state of the nanoencapsulated enzyme. We recorded the CD spectral changes in the aromatic absorption range produced by GlcNAc6P binding by using ligand-free monoliths in either quaternary conformation.

**Figure 5:**
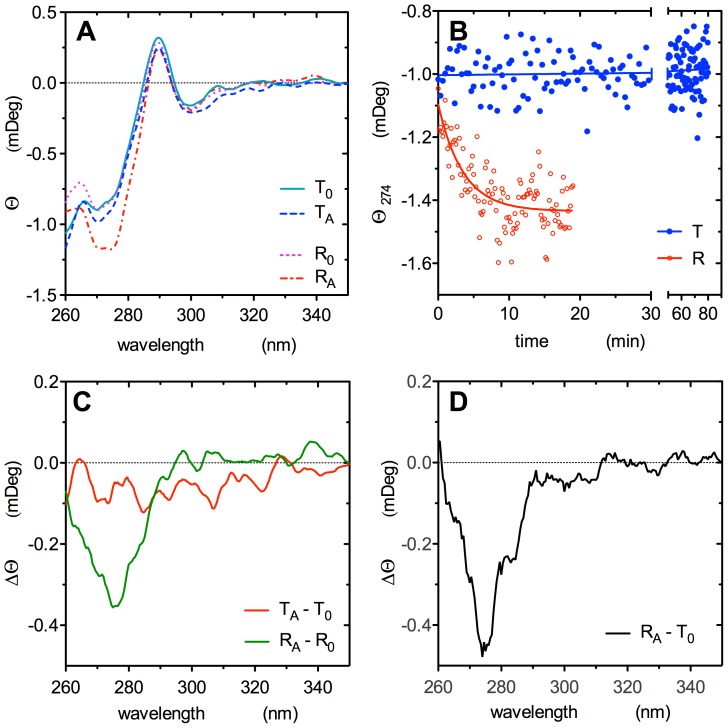
Circular Dichroism spectra of GNPDA-doped gels in the aromatic absorption range. (A) continuous line, CD spectrum of a monolith doped with ligand-free enzyme in T conformer; dashed line, spectrum of the same T gel equilibrated with 1 mM GlcNAc6P; dotted line, CD spectrum of a monolith doped with ligand-free enzyme in R conformer; dotted-dashed line, spectrum of the same R gel equilibrated with 1 mM GlcNAc6P. (B) Time course of CD change at 274 nm. The ligand-free enzyme nanoencapsulated in either quaternary conformer was added with 1 mM GlcNAc6P: (•), T form; (○), R form. The decay curve given by the R conformer was fitted to a simple exponential function. (C) Differential CD spectrum between the activator-saturated conformers and its correspondent ligand-free forms, showing the ellipticity change of the nanoencapsulated enzyme produced by GlcNAc6P binding. Red line: T_A_-T_0_; green line, R_A_ - R_0_. (D) CD spectral difference between the activator-saturated R doped gel and the T_0_ gel. These forms of the enzyme are equivalent to the extreme states observed in solution (T_0_ and R_A_); this differential spectrum matches with the corresponding spectral changes reported in solution [34].

Figure 5A shows the near-UV CD spectra, obtained with T or R-state nanoencapsulated enzyme, both as ligand-free and GlcNAc6P-saturated. The previously identified spectral change associated with allosteric activation in solution is absent in either ligand-free quaternary conformers. Upon the addition of a saturating concentration of the allosteric activator, the monoliths doped with T_0_ enzyme did not present the ellipticity change at 274 nm. Actually, the ellipticity of the T form at this wavelength remains stable for at least 80 min (Figure 5B), in spite that by this time the enzyme becomes allosterically activated (Figure 2). Only the enzyme in the R state when in presence of the activator, displays the characteristic spectral change of the enzyme-GlcNAc6P complex in solution (Figure 5C). Figure 5B shows the time course of this spectral change, recorded after the addition of a saturating concentration of the activator to the nanoencapsulated R_0_ form. The differential CD spectrum between the free and the activator-bound forms in solution [17] shows a similar profile to the differential spectrum of the nanoencapsulated forms, R_A_ against T_0_, verifying that the nanoencapsulated enzyme yields equivalent results as the enzyme in solution (Figure 5D).

**Figure 6:**
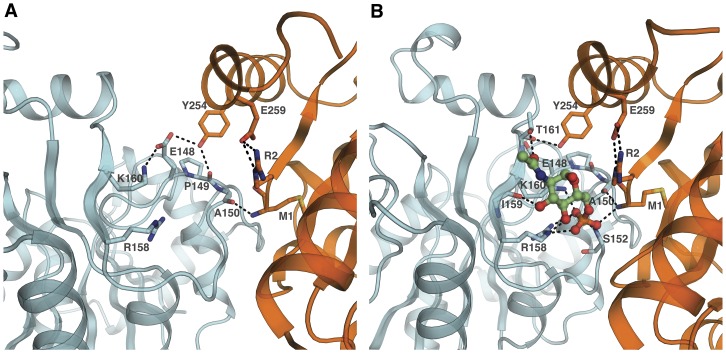
EcGNPDA allosteric site represented at each quaternary conformation. (A) The intersubunit cleft in the T state (PDB 1FSF) and (B) in the R state (PDB 1FS5) [14]. Face A is colored in blue and face NC in orange; GlcNAc6P carbons are in green. The allosteric site in both quaternary conformations was oriented with respect to face C to emphasize its changes in relation to face A. The figure shows the cleft between faces A and NC where GlcNAc6P binding site is located, this space narrows on quaternary transition from T to R state; its displacement is blocked in the T gel. In spite of this apparent disruption of the GlcNAc6P binding site, the nanoencapsulated T state enzyme still binds the activator. The shown interactions of this ligand to face A in the R state must be available also in the T conformation. The GlcNAc6P interaction in the face A of the T state can trigger a tertiary activation restricted to its own subunit. The interfacial contacts disrupted in T state must play a critical role in the propagation of cooperativity to the whole hexamer. Images were prepared using PyMOL [37].

The CD spectral change at 274 nm originates in the interactions built by the hydroxyl phenolic oxygen of Tyr254 with the neighboring polypeptide chain of the vicinal subunit [19], [34]. Tyr254 was already recognized as the origin of this CD reporter signal, because the site-directed Tyr254-Phe mutant does not present this negative CD peak change [19]. These results, taken together, confirm the tertiary origin of the allosteric activation and its independence on the quaternary background of the molecule. This spectral signal detects the occupation state of the allosteric site exclusively in the R quaternary background. The absence of some critical intersubunit contact displacements is reflected on the differential CD spectra given by T_A_r and R_A_r complexes (Figure 5C). Only the tertiary *r*-state in an R quaternary background can build these interfacial contacts on the allosteric site, including those involving Tyr254 phenolic hydroxyl group (Figure 6). This property helps to assert the quaternary state in which the enzyme is trapped and demonstrates that the allosteric activation of the nanoencapsulated T conformer occurs independently of the quaternary change and does not involve modifications of the interfacial subunit contacts.

### Structural implications of the dissociation of allosteric activation and cooperativity in the nanoencapsulated EcGNPDA

Previous crystallographic and physicochemical studies indicated the existence of conformational sub-states of the R conformer of EcGNPDA, [14], [21] however, its T state remained unexplored from this point of view, mainly due to the short-lived condition of the involved intermediates. Nanoencapsulation of the quaternary-locked conformers of the enzyme resulted particularly suitable for the study of the T state. We have found that GlcNAc6P activates the gel-trapped enzyme with similar catalytical parameters, such as those reported for the enzyme in aqueous phase (Tables 1 and 2). This implies that the enzyme is capable of building all the required interactions with bound GlcNAc6P in order to stabilize the occupied subunit in the high affinity tertiary state. The activation by GlcNAc6P of the subunits in a T background is similar to the activation of the soluble enzyme (Tables 1 and 2). However, the allosteric site presents drastic structural differences when comparing the crystallographic structures of both allosteric conformers. The allosteric site is formed in a cleft between neighboring subunits around the three-fold axis of symmetry of the hexamer (Figure 6). Both sides of the allosteric site in the R conformer have binding contacts with the activator molecule, while the allosteric site of the ligand-free T state appears drastically modified. Our results indicate that under a T quaternary conformation, probably not too different to that shown in Figure 6A, individual subunits are able to undergo a tertiary transition in the presence of GlcNAc6P and build a high-affinity binding site for the substrate. Face A of the allosteric site (Figure 6) is contiguous to the active site and contains most of the GlcNAc6P binding contacts. This is most likely the only face involved in the activator binding in the T state. The other face, containing the N and C-terminal segments of the molecule (face NC in Figure 6), shows two intersubunit interactions that are modified by the quaternary transition and whose role in promoting cooperativity has been demonstrated. [18], [19]. These interactions involve two key groups on face NC, both forming contacts with face A. The phenolic hydroxyl group of Tyr254 that forms a direct intersubunit interaction and the N-terminal amino group (face NC) interacting with face A through the phosphate bridge formed by the 6-phospho group of bound GlcNAc6P. The interruption of these contacts was brought about by site-directed mutagenesis or specific chemical modification of N-terminal amino group, respectively. It is worth to remark that these changing contacts of Tyr254 are the origin of CD spectral changes associated with the T-R transition. Both modifications produced velocity *versus* substrate patterns at different GlcNAc6P concentrations that resemble those of the allosteric activation of the trapped T conformer.

On the facing side of the allosteric site, some intrasubunit changes involving the active and allosteric sites have been described. Even if we do not know the structural details of the *t-r* transition, some changes observed in the crystallographic structures can be considered as possible components of the intrasubunit conformerization. A striking example is the rotation of the Glu148 side chain that interacts with the catalytic His143 in the R structure and moves in the T state, forming a salt-link with Lys160, on the allosteric site of the same face A [13]. While tertiary transition under a T background only modifies GlcNAc6P binding properties of face A, the interfacial interaction appearing in the R state seems to be responsible for coupling the tertiary transitions with the quaternary rearrangement of the whole hexamer.

## Concluding Remarks

We characterized the allosteric activation of the nanoencapsulated T conformer of EcGNPDA under conditions of total suppression of the quaternary transition. Activation by GlcNAc6P is elicited by its binding to face A of each subunit (Figure 6), which undergoes a conformational change. These changes can be interpreted as a transition between two extreme tertiary states *t* and *r*, respectively, of low and high affinities for its ligands, GlcN6P and GlcNAc6P. Our analyses of GlcNAc6P binding revealed the sequential nature of this conformational change that modifies the binding properties of the active site located in the same subunit. We provided kinetic evidence for the existence of tertiary mixed forms at EcGNPDA T conformers. Our results pointed out the role of the intersubunit contacts between faces A and NC at the allosteric site, in the coupling mechanism of tertiary events to the quaternary T-R transition, which trigger homotropic cooperativity in the free enzyme in solution. The present studies on the T conformer of EcGNPDA shed some light on its coupling mechanism and explain the easiness of turning *K*-type activation into *V*-type one by modification of intersubunit contacts. This tertiary-quaternary linkage was firstly described for hemoglobin [23]. It is remarkable that two allosteric proteins without a close evolutionary relationship, such as mammalian hemoglobin and EcGNPDA are so similar in the mechanism linking allosteric effects and cooperativity. The coherence of our results with the postulates of the Tertiary Two-States (TTS) model proposed for hemoglobin [36], is appealing.

## Acknowledgments

We thank Prof. Dr. Rosario Muñoz-Clares for her critical comments and many fruitful discussions. We are grateful to Prof. Dr. Andrea Mozzarelli for helpful and encouraging discussions and Dr. Barbara Pioselli for helping us in setting up nanoencapsulation conditions for EcGNPDA. We are indebted to Perla Ovseiovich from *Paralelo 19* for help on the striking image design.
